# Dibutyl Phthalate (DBP)-Induced Apoptosis and Neurotoxicity are Mediated via the Aryl Hydrocarbon Receptor (AhR) but not by Estrogen Receptor Alpha (ERα), Estrogen Receptor Beta (ERβ), or Peroxisome Proliferator-Activated Receptor Gamma (PPARγ) in Mouse Cortical Neurons

**DOI:** 10.1007/s12640-016-9665-x

**Published:** 2016-08-31

**Authors:** Anna K. Wójtowicz, Konrad A. Szychowski, Agnieszka Wnuk, Małgorzata Kajta

**Affiliations:** 1 Department of Animal Biotechnology, Animal Sciences Faculty, University of Agriculture, Redzina 1B, 30-248 Kraków, Poland; 2 Department of Public Health, Dietetics and Lifestyle Disorders, Faculty of Medicine, University of Information Technology and Management in Rzeszow, Sucharskiego 2, 35-225 Rzeszow, Poland; 3 Department of Experimental Neuroendocrinology, Institute of Pharmacology, Polish Academy of Sciences, Smetna 12, 31-343 Kraków, Poland

**Keywords:** DBP, Apoptosis, ERα, ERβ, PPARγ, AhR, Phthalate, Neuron

## Abstract

Dibutyl phthalate (di-*n*-butyl phthalate, DBP) is one of the most commonly used phthalate esters. DBP is widely used as a plasticizer in a variety of household industries and consumer products. Because phthalates are not chemically bound to products, they can easily leak out to enter the environment. DBP can pass through the placental and blood–brain barriers due to its chemical structure, but little is known about its mechanism of action in neuronal cells. This study demonstrated the toxic and apoptotic effects of DBP in mouse neocortical neurons in primary cultures. DBP stimulated caspase-3 and LDH activities as well as ROS formation in a concentration (10 nM–100 µM) and time-dependent (3–48 h) manner. DBP induced ROS formation at nanomolar concentrations, while it activated caspase-3 and LDH activities at micromolar concentrations. The biochemical effects of DBP were accompanied by decreased cell viability and induction of apoptotic bodies. Exposure to DBP reduced *Erα* and *Pparγ* mRNA expression levels, which were inversely correlated with protein expression of the receptors. Treatment with DBP enhanced *Ahr* mRNA expression, which was reflected by the increased AhR protein level observed at 3 h after exposure. ERα, ERβ, and PPARγ antagonists stimulated DBP-induced caspase-3 and LDH activities. AhR silencing demonstrated that DBP-induced apoptosis and neurotoxicity are mediated by AhR, which is consistent with the results from DBP-induced enhancement of AhR mRNA and protein expression. Our study showed that AhR is involved in DBP-induced apoptosis and neurotoxicity, while the ERs and PPARγ signaling pathways are impaired by the phthalate.

## Introduction

Dibutyl phthalate (di-*n*-butyl phthalate, DBP) is one of the most commonly used phthalate esters and is an endocrine-disrupting chemical (EDC). DBP is widely used as plasticizer in a variety of household industries and consumer products, such as toys, food containers, furniture, cosmetics and personal care products, latex adhesives, cellulose plastics, varnishers, and dye solvents (Stales et al. 1997; Shea 2003; Duty et al. 2005; Heudorf et al. 2007). Because phthalates are not chemically bound to products, they can easily diffuse within the materials and enter the environment (Fromme et al. 2002; Fujii et al. 2003). Several studies have evaluated the DBP concentrations in the air and dust of residences and found that DBP could reach detrimentally high levels (Rudel et al. 2003; Adibi et al. 2003; Fromme et al. 2004; Otake et al. 2004). Phthalates are rapidly absorbed after inhalation or oral administration due to their lipophilic properties (Rudel et al. 2003; Adibi et al. 2003; Kavlock et al. 2006). DBP has been found in various tissues, despite the fact that it is rapidly hydrolyzed to monobutyl phthalate by non-specific lipases and esterases (Rowland et al. 1977; Tanaka et al. 1978; Foster et al. 1983). Elevated DBP concentrations have been found in human cord blood (68.14 µg/L = 244.8 nM), milk (42 ng/mL = 150.89 nM), blood (9.1 ng/mL = 32.69 nM), and urine (38 ng/mL = 136.52 nM) (Högberg et al. 2008; Huang et al. 2014). A study conducted on male Wistar rats showed that DBP was detected in rat brains after a single oral dose (Williams and Blanchfield 1975). Furthermore, DBP accumulation was much higher after chronic (3 or 6 months) exposure than after a single inhalation (Kawano 1980), thus indicating effective accumulation of the phthalate in brain tissue. DBP has also been shown to pass through the placental and blood–brain barriers in rats (Williams and Blanchfield 1975; Kawano 1980; Saillenfait et al. 1998; Huang et al. 2014). However, little is known about mechanisms of action of DBP in the nervous system, especially in the early developmental stages.

A recent report showed that DBP caused deleterious effects in the developing nervous system. This compound inhibited acetylcholinesterase activity in zebrafish (*Danio rerio*) embryos and up-regulated transcripts of growth-associated protein 43, embryonic lethal abnormal vision-like 3, glial fibrillary acidic protein, myelin basic protein, α1-tubulin and neurogenin1 (Xu et al. 2013). DBP also had adverse effects on the behavior and cognitive abilities of rats that were prenatally exposed to the phthalate (Li et al. 2009). Recently, exposures to DBP have been correlated with behavioral disorders in eight-year-old children (Lien et al. 2014). Moreover, Li et al. (2013, 2014) found altered expression of aromatase, estrogen beta receptor (ERβ), brain-derived neurotrophic factor, and p-CREB as well as cytotoxicity and apoptosis in the hippocampal neurons of DBP-exposed immature rats. Extensive reactive oxygen species (ROS) production and oxidative DNA damage were detected in rat livers, kidneys, and testes following exposure of rats to DBP (Wellejus et al. 2002). Oxidative stress was recently shown to have a critical role in DBP-induced neurotoxicity in *Caenorhabditis elegans* (Tseng et al. 2013). DBP is capable of binding to nuclear receptors, such as estrogen receptor alpha (ERα), ERβ, peroxisome proliferator-activated receptor gamma (PPARγ), and aryl hydrocarbon receptor (AhR), in various tissues. However, there is almost no data on the involvement of these receptors in the DBP-mediated effects in neural tissues. Although apoptotic and neurotoxic effects of DBP have been identified, its function as an EDC has only been partially characterized. Studies on the involvement of ERs and xenobiotic receptors in DBP-mediated effects are needed.

The aim of the present study was to investigate the cytotoxic and apoptotic effects of DBP and its influence on ROS production in mouse neocortical neurons 3–48 h after exposure. To explore the molecular mechanisms of DBP action on neocortical neurons, we studied the involvement of ERα, ERβ, PPARγ, and AhR in DBP-induced effects.

## Materials and Methods

### Reagents

Neurobasal medium without phenol red and B27-AO supplements, and the TaqMan probes corresponding to specific genes encoding β-actin (Mm00607939_s1), AhR (Mm01291777_m1), Esr1 (Mm00433147_m1), Esr2 (Mm01281854_m1), and PPARγ (Mm00440945_m1) were purchased from Life Technologies (Grand Island, NY, USA). Estradiol (ER agonist), α-naphthoflavone (αNF, AhR antagonist), β-naphthoflavone (βNF, AhR agonist), GW1929 (PPARγ agonist), GW9662 (PPARγ antagonist), trypsin, charcoal/dextran-treated fetal bovine serum (FBS), penicillin, streptomycin, glycerol, Tris, HEPES, CHAPS, DTT, Nonidet NP-40, SDS, EDTA, Tween 20, 2,7′-dichlorodihydrofluorescein diacetate (H_2_DCFDA), bromophenol blue, staurosporine, Hoechst 33342, calcein AM, anti-β-actin antibody (A5316), DMSO, and DBP (524980) were purchased from Sigma-Aldrich (St. Louis, MO, USA). Caspase-3 substrate was purchased from Merck (Darmstadt, Germany). An ERα antagonist, 1,3-bis(4-hydroxyphenyl)-4-methyl-5-[4-(2-piperidinylethoxy)phenol]-1h-pyrazole dihydrochloride (MPP), and an ERβ antagonist, 4-[2-phenyl-5,7-bis(trifluoromethyl)pyrazolo[1,5-a]pyrimidin-3-yl]phenol (PHTPP), were purchased from Tocris Bioscience (Bristol, United Kingdom). The cytotoxicity detection kit was purchased from Roche Applied Science (Munich, Germany). INTERFERin^®^ siRNA transfection reagent was purchased from Polyplus-transfection (Illkirch, France). AhR siRNA (sc-29655), ERα siRNA (sc-29306), ERβ siRNA (sc-35326), PPARγ siRNA (sc-29456), and anti-AhR (sc-8088), anti-ERα (sc-7207), anti-ERβ (sc-8974), and anti-PPARγ (sc-7273) antibodies were purchased from Santa Cruz Biotechnology, Inc. (Santa Cruz, CA, USA). The Bio-Rad protein assay was purchased from Bio-Rad Laboratories (Munich, Germany). Stock solutions of the test compounds were prepared in DMSO and were added to the neurobasal medium. The final concentration of DMSO in the culture medium was always 0.1 %.

### Primary Cultures of Neocortical Neurons

The experiments were performed using primary cultures of mouse cortical neurons. These cultures were prepared from the fetuses of pregnant female Swiss mice as previously described (Brewer 1997; Szychowski et al. 2015). Brain tissues were collected from the mouse embryos on day 15 and 16 of gestation. Pregnant females were anesthetized with CO_2_ vapor and killed by cervical dislocation. The animal care protocols were in accordance with official governmental guidelines, and all efforts were made to minimize the number of animals used and their suffering. All procedures were performed in accordance with the National Institutes of Health Guidelines for the Care and Use of Laboratory Animals and were approved by the Bioethics Commission (No. 83/2012), in compliance with Polish law. The brains were removed from the fetuses, and the cortical tissues were dissected. The dissected tissue was minced into small pieces and then gently digested with trypsin. Then, the cells were centrifuged, and the pellet was resuspended in phenol red-free neurobasal medium supplemented with 5 % charcoal/dextran-treated fetal bovine serum. The cells were plated onto poly-l-ornithine-coated (0.01 mg/mL) multi-well plates. After 2 days, the culture medium was changed to neurobasal medium supplemented with B27-AO (2 µL/mL), glutamine (2 mM), 50 U/mL penicillin, and 0.05 mg/mL streptomycin, which is recommended for primary neuronal cultures (Brewer 1997; Kajta et al. 2005). For the experiments, the cells were cultured at a density of 1.8 × 10^5^ cells/cm^2^. This procedure typically yields cultures that contain approximately 90 % neurons and 10 % astrocytes (Kajta et al. 2004). The cultures were maintained at 37 °C in a humidified atmosphere containing 5 % CO_2_ and were cultivated for 7 days in vitro prior to the experiment. The culture medium was changed prior to treating the cultures with the compounds selected for this study. Experimental concentrations were chosen according to the literature data. The concentrations used by other authors were in a range between 0.61 µM in SH-SY5Y cells, 1–100 µM in rat neural stem cells, and 200 µM in rat explants of cerebellum (Kasuya 1974; Kaun-Yu et al. 2004; Ishido and Suzuki 2014).

### siRNA Gene Silencing

Specific siRNAs targeting ERα, ERβ, PPARγ, and AhR were used to inhibit gene expression in mouse neocortical neurons using a previously described method with modifications (Kajta et al. 2014). The siRNA was applied for 7 h at a final concentration of 50 nM in antibiotic-free medium containing the siRNA transfection reagent interferin. After transfection, the culture media were changed, and the neurons were cultured for 24 h before starting the experiment. Controls included positive siRNA and negative siRNA containing a scrambled sequence that did not lead to the specific degradation of any known cellular mRNA. The effectiveness of mRNA silencing was verified by measurement of specific mRNAs with real-time PCR and Western blot analysis and by selective ligand binding to the receptor (results not shown).

### Measurement of ROS

The fluorogenic dye 2,7′-dichlorodihydrofluorescein diacetate (H_2_DCFDA) was used to detect intracellular ROS. After diffusion into the cell, H_2_DCFDA is deacetylated by cellular esterases into a non-fluorescent compound that is subsequently oxidized by ROS into 2,7′-dichlorofluorescein (DCF) (Gomes et al. 2005). ROS levels were measured with 5 µM H_2_DCFDA to determine the ability of DBP to induce ROS production in the neocortical neurons. For the ROS measurements, the cells were plated onto black-sided, clear-bottomed 96-well plates and exposed to 10 nM to 100 µM of DBP for 3, 6, and 24 h. The cells were incubated with H_2_DCFDA in serum-free and phenol red-free neurobasal medium for 45 min before DBP treatment. After 3, 6, and 24 h of incubation of the cells with DBP (5 % CO_2_ at 37 °C), the culture medium was replaced with fresh neurobasal medium to remove extracellular residual DCF and DBP to reduce the fluorescence background. Cells treated with 55 µM tert-butyl hydrogen peroxide were used as a positive control (results not shown). The interaction between DBP and H_2_DCFDA was tested in cell-free conditions before the experiments (results not shown) to address the concerns about the H_2_DCFDA assay previously described by Szychowski and Wójtowicz (2016). DCF fluorescence was detected using a microplate reader (Bio-Tek FLx800) at maximum excitation and emission spectra of 485 and 535 nm, respectively. The data were analyzed using KCJunior software (Bio-Tek Instruments) and were normalized to the fluorescence in the vehicle-treated cells. The results are expressed as the mean percent of the control from eight separate samples ± SEM, and the samples were tested in quadruplicate.

### LDH Cytotoxicity Assay

The cytotoxicity detection kit is a colorimetric assay for the quantification of cell death and cell lysis based on the release of lactate dehydrogenase (LDH) from the cytosol of damaged cells into the supernatant (Koh and Choi 1987). An increase in the amount of dead or plasma membrane-damaged cells results in an increase in LDH release in the culture supernatant. Primary neocortical cell cultures were exposed to increasing concentrations (10, 50, 100 nM and 10, 25, 50, 100 μM) of DBP. After the cells were cultured in 96-well plates, 100 µL of the medium was collected for the LDH analysis, and the cells were collected and frozen at −80 °C for measurement of the caspase-3 activity. Control (no vehicle) and DMSO-treated samples were included in the experimental design to determine the effects of DMSO (results not shown). For the LDH assay, 100 µL of the collected supernatant was incubated with the reaction mixture provided in the LDH assay kits. After 30 min, the reaction was stopped by adding 1 N HCl, and the absorbance was measured at a wavelength of 490 nm with a reference wavelength of 600 nm in a micro-ELISA plate reader. The data were analyzed using KCJunior software (Bio-Tek Instruments) and were normalized to the fluorescence in the vehicle-treated cells. The results are expressed as the mean percent of the control from eight separate samples ± SEM, and the samples were assayed in quadruplicate.

### Measurement of Caspase-3 Activity

Caspase-3 activity was used as a marker for cell apoptosis and was determined using the method described by Nicholson et al. (1995). After thawing (−80 °C), neurons were lysed using lysis buffer (50 mM HEPES, pH 7.4, 100 mM NaCl, 0.1 % CHAPS, 1 mM EDTA, 10 % glycerol, 10 mM DTT). The lysates were incubated with the specific substrate for caspase-3, Ac-DEVD-pNA, at 37 °C. Cells treated with 1 µM staurosporine were used as a positive control (results not shown). After 30 min, the absorbance of the lysates was measured at 405 nm in a microplate reader (Bio-Tek ELx800). The formation of the colorimetric product was continuously monitored for 120 min. The data were analyzed using KCJunior (Bio-Tek Instruments) and normalized to the absorbance of the vehicle-treated cells. The results are expressed as the mean percent of the control from eight separate samples ± SEM, and the samples were assayed in quadruplicate.

### Calcein AM Staining

The esterase activities of living cells are visualized by calcein AM as green fluorescence. Therefore, this staining protocol was used to assess the metabolism and cell viability (Kajta et al. 2009). For calcein AM staining, neurons were seeded on polyornithine-coated coverslips in 24-well plates and cultured in the presence of 10 μM of DBP for 24 h. The cells were washed with PBS to eliminate the esterase activity present in the growth media,. The cells grown on glass cover slips were then incubated in 4 µM calcein AM in PBS at 37 °C in a 5 % CO_2_ atmosphere for 10 min. Cells with bright yellow cytoplasm were identified as living cells. Fluorescence microscopy (Nikon, Japan) was used to visualize the stained cells.

### Identification of Apoptotic Cells with Hoechst 33342 Staining

Apoptotic cells show nuclear condensation and DNA fragmentation, which are detected by Hoechst 33342 staining. Hoechst 33342 binds the DNA fragments and the apoptotic bodies, emitting blue fluorescence (Kajta et al. 2009). For Hoechst 33342 staining, neurons were seeded on polyornithine-coated coverslips in 24-well plates. After an initial treatment with 10 µM of DBP for 24 h, the cells were washed with PBS and incubated with Hoechst 33342. Hoechst 33342 was diluted with PBS and added to the medium at a final concentration of 10 μM. The cells were incubated for 10 min in a 5 % CO_2_ atmosphere at 37 °C and then visualized with a fluorescence microscope (Nikon, Japan).

### Western Blot Analysis

For the estimation of protein expression, neurons were cultured on polyornithine-coated 6-well plates in the presence of 10 μM of DBP for different time intervals (0, 1, 3, 6, 24, and 48 h). The cells were lysed in 100 µL of ice-cold lysis buffer containing 100 mM NaCl, 50 mM Tris HCl (pH 7.5), 0.5 % Na-deoxycholate, 0.5 % Nonidet NP-40, and 0.5 % SDS. Then, the lysates were sonicated and clarified by centrifugation at 15,000×*g* at 4 °C for 20 min, and the supernatant was collected and stored at −80 °C until analysis. The protein concentrations in the supernatants were determined with the Bradford method (Bradford 1976) using bovine serum albumin as the standard. From the whole cell lysates, 35 µg of total protein was added to an appropriate amount of sample buffer consisting of 125 mM Tris (pH 6.8), 4 % SDS, 25 % glycerol, 4 mM EDTA, 20 mM DTT, and 0.01 % bromophenol blue. Samples were separated by 7.5 % SDS–polyacrylamide gel electrophoresis in a Bio-Rad Mini-Protean II electrophoresis cell, and the proteins were then transferred to nitrocellulose membranes using a Bio-Rad Mini Trans-Blot apparatus. Following the transfer, the membranes were washed, and non-specific binding sites were blocked with 5 % dried milk and 0.2 % Tween 20 in 0.02 M TBS for 2 h. Then, the membranes were incubated overnight with the anti-PPARγ, anti-ERα, anti-ERβ, and anti-AhR antibodies diluted 1:200 in TBS/Tween at 4 °C. After incubation with the primary antibodies, the membranes were washed with TBS and 0.02 % Tween 20 and incubated for 2 h with horseradish peroxidase-conjugated secondary antibodies diluted 1:500 in TBS/Tween. β-Actin was used as a loading control with an anti-β-actin antibody diluted 1:3000 in TBS/Tween (secondary antibody diluted at 1:5000 in TBS/Tween). Signals were detected by chemiluminescence using western blotting luminol reagent and visualized with a Fuji LAS-4000 phosphorimager. The intensities of the immunoreactive bands were quantified by densitometry. Densitometry was performed using ImageJ 1.47v software (National Institutes of Health, USA).

### Real-Time PCR Analysis of PPAR-γ, ERα, ERβ, and AhR

Total RNA was extracted from neocortical neurons exposed to 10 µM of DBP for 3 or 6 h using a previously described method (Kajta et al. 2014). A Qiagen RNeasy mini kit was used for extraction according to the manufacturer’s protocol. The quantity of RNA was determined spectrophotometrically at 260 and 280 nm (ND-1000 UV–Vis; Thermo Fisher NanoDrop, USA). Two-step real-time RT-PCRs were performed. Both the reverse transcription (RT) reaction and the quantitative polymerase chain reaction (qPCR) were conducted using the CFX96 Real-Time System (Bio-Rad, USA). The RT reaction had a final volume of 20 μL with 300 ng of RNA (as a cDNA template) using a cDNA reverse transcription kit according to the manufacturer’s protocol. Products of the RT reaction were amplified with the TaqMan Gene Expression Master Mix (Life Technologies Applied Biosystems, USA) kit using TaqMan probes as primers for the specific genes encoding β-actin, ERα, ERβ, PPARγ, and AhR. Amplification was carried out in a total volume of 20 μL containing 1x TaqMan Gene Expression Master Mix and 1 μL of RT product used as the PCR template. The standard qPCR reaction was performed as follows: 2 min at 50 °C and 10 min at 95 °C followed by 40 cycles of 15 s at 95 °C and 1 min at 60 °C. The threshold value (Ct) for each sample was determined during the exponential phase, and the ΔΔCt method was used for data analysis. β-actin was used as the reference gene.

### Statistical Analysis

Data are presented as the mean ± SEM of four independent experiments. Each treatment was repeated eight times (*n* = 8) and assayed in quadruplicate; thus, the total number of replicates was 32. The average of the quadruplicate samples was used for the statistical calculations. Data were analyzed by one-way analysis of variance followed by Tukey’s multiple comparison test. Differences between the control and experimental groups are indicated as follows: *p < 0.05, **p < 0.01, ***p < 0.001 versus control cells in all experiments, ###*p* < 0.001 versus the cells transfected with the negative siRNA (Figs. 6, 7), #*p* < 0.05, ###*p* < 0.001, cells treated with 10 µM of DBP versus the cells treated with 10 µM of DBP with co-administration of a receptor antagonist (Fig. 8).

## Results

### DBP-Stimulated ROS Production

**Fig. 1:**
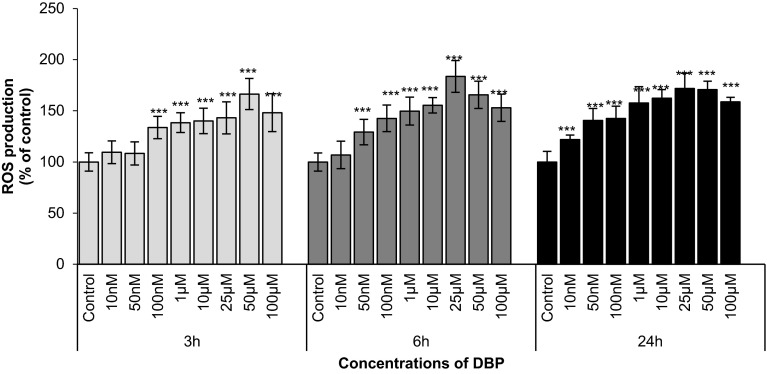
The effects of increasing concentrations of DBP (10, 50, and 100 nM and 1, 10, 25, 50, and 100 μM) on ROS formation in cultured neocortical neurons after 3, 6, and 24 h of exposure. Each point represents the mean ± SEM of four independent experiments, each of which consists of eight replicates per treatment group. ***p < 0.001 versus the control cultures

Neocortical neuron cultures were treated with DBP concentrations ranging from 10 nM to 100 μM. After 3 h of exposure, the ROS production increased by 34–66 % compared with that of the vehicle control. Increased ROS production was also observed after 6 and 24 h of treatment, with DBP increasing ROS by 22–84 % of that of the control (Fig. 1). The capacity of DBP to stimulate ROS production increased with the duration of exposure. At 3 h of exposure, only concentrations of 100 nM DBP or higher stimulated ROS formation at 6 h of exposure, concentrations of 50 nM DBP or higher stimulated ROS formation and at 24 h of exposure, DBP was already effective at a concentration of 10 nM.

### DBP-Induced Effects on LDH Release and Cell Viability

**Fig. 2:**
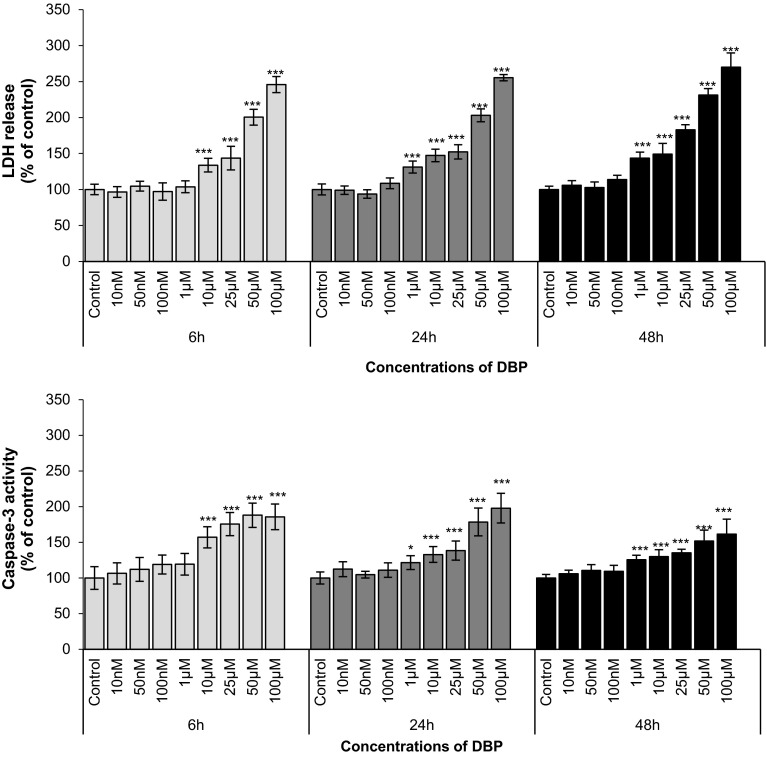
The effects of increasing concentrations of DBP (10, 50, and 100 nM and 1, 10, 25, 50, and 100 μM) on LDH release and caspase-3 activity in cultured neocortical neurons after 6, 24, and 48 h of exposure. Each point represents the mean ± SEM of four independent experiments, each of which consisted of eight replicates per treatment group. *p < 0.05, **p < 0.01, ***p < 0.001 versus the control cultures

DBP at concentrations of 10 µM or higher (25, 50, 100 μM) caused a substantial 34–146 % increase in LDH release compared with that of the vehicle control after 6 h. Similar effects were observed after 24 and 48 h of exposure to DBP. However, during prolonged exposures to DBP, LDH release increased even with a concentration of 1 µM DBP (Fig. 2a).

**Fig. 3:**
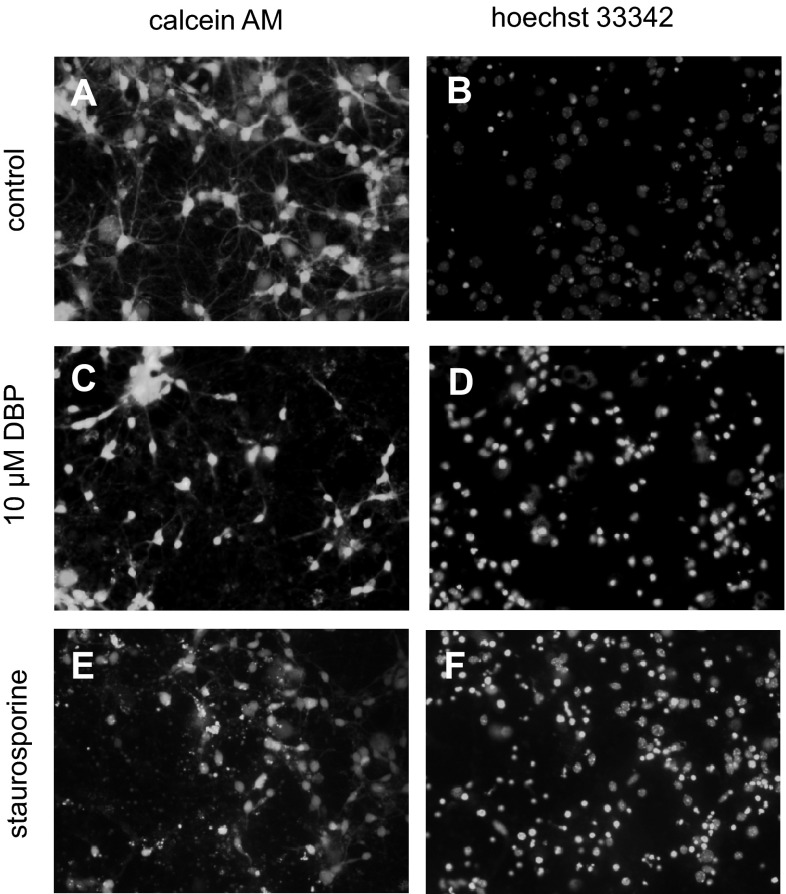
Effects of DBP on Hoechst 33342 and calcein AM staining in cultures of neocortical neurons examined 24 h post treatment. **a** Control cells stained with calcein AM. **b** Control cells stained with Hoechst 33342. **c** Cells treated with 10 μM DBP and stained with calcein AM. **d** Cells treated with 10 μM DBP and stained with Hoechst 33342. **e** Cells treated with 1 μM staurosporine and stained with calcein AM. **f** Cells treated with 1 μM staurosporine and stained with Hoechst 33342. Cells with bright yellow fluorescence were identified as live cells. Cells with bright, fragmented nuclei containing condensed chromatin were identified as apoptotic cells. Photomicrographs are shown at ×200

Neurons were stained with calcein AM to assess the viability of the cells. Living cells display green fluorescence. In the control culture, healthy cells with green fluorescence predominated (Fig. 3). After 24 h of exposure to 10 μM DBP, a reduction in green fluorescence was observed. Staurosporine (1 µM) was used as a positive control and caused massive cell death.

### Apoptotic Effect of DBP

Caspase-3 activity increased significantly following a 6 h treatment with 10, 25, 50, and 100 μM DBP. The activity was increased in DBP-treated neuron cultures by 57–88 % of the activity of the vehicle control (Fig. 2b). Enhanced enzyme activity was also detected after 24 and 48 h exposures to the phthalate. In these prolonged exposures, DBP was effective even at the lower concentration of 1 µM.

Neurons were stained with Hoechst 33342 to assess apoptosis. Apoptotic bodies appeared as bright blue fragmented nuclei that showed condensed chromatin, which is characteristic of apoptotic cells. In the control culture, healthy cells with intact nuclei were predominant (Fig. 3). The apoptotic bodies were detected after 24 h of exposure to 10 μM of DBP. In addition, staurosporine (1 µM) induced apoptotic bodies in neocortical cells.

### Effect of DBP on the mRNA Expression of *ERα*, *ERβ, PPARγ*, and *AhR*

**Fig. 4:**
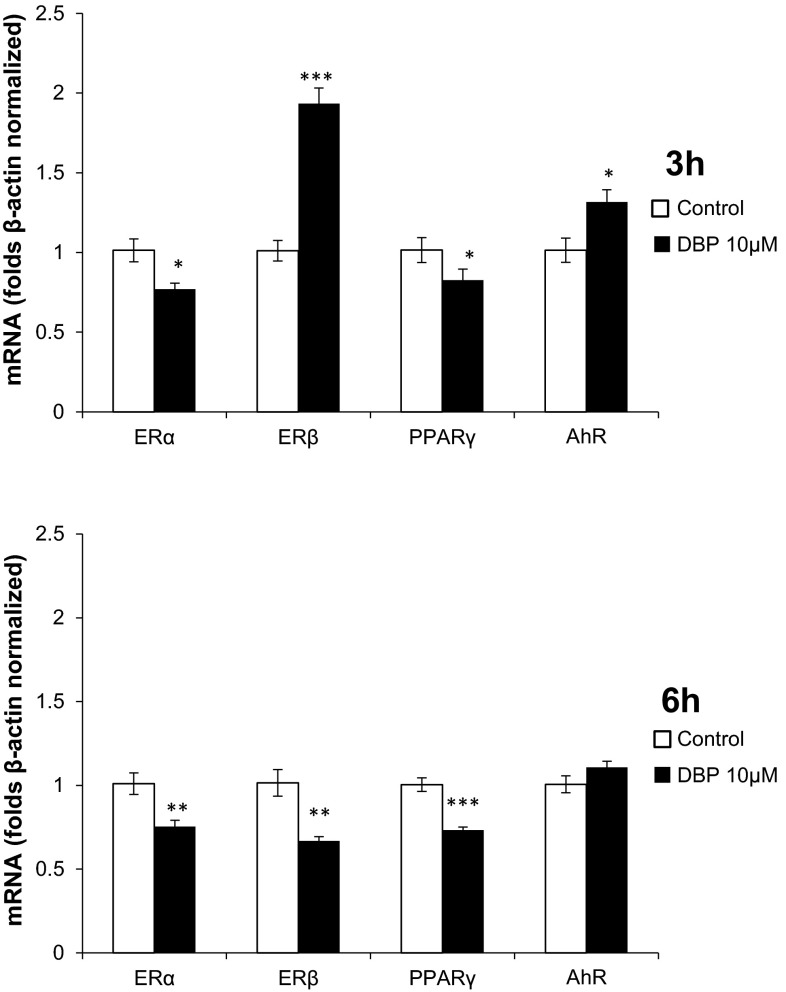
The effect of 10 µM of DBP on mRNA expression of *ERα*, *ERβ*, *PPARγ*, and *AhR* after 3 h (**a**) and 6 h (**b**) of exposure. mRNA expression was normalized to β-actin expression. The data are expressed as the mean ± SEM of four independent experiments, each of which consisted of eight replicates per treatment group. *p < 0.05, **p < 0.01, ***p < 0.001 versus the control

Neocortical neurons were exposed to DBP (10 μM) for 3 h, and a decrease in the mRNA expression of *ERα* and *PPARγ* (decreased by 24.39 and 18.86 %, respectively) compared to that of the control was observed. However, there was an increase in the expression of *ERβ* and *AhR* compared to that of the control (increased by 92.38 and 30.23 %, respectively) (Fig. 4a).

After the cells were exposed to 10 µM of DBP for 6 h, we observed decreased mRNA expression of *ERα*, *ERβ*, and *PPARγ* compared to that of the control (decreased by 25.60, 34.60, and 27.17 % respectively) (Fig. 4b).

### Effect of DBP on Protein Expression of PPARγ, AhR, ERα, and ERβ

**Fig. 5:**
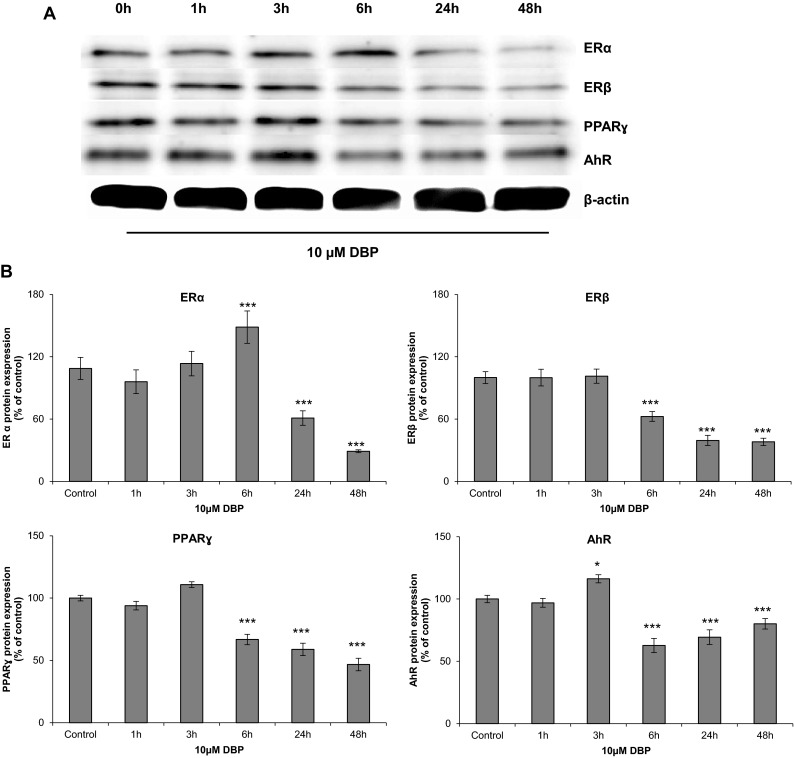
Representative western blots of ERα, ERβ, PPARγ, and AhR proteins in neocortical neurons treated with 10 μM of DBP after 1, 3, 6, 24, and 48 h (**a**). Protein bands were quantified by densitometry. The results are shown as the percentage of ERα, ERβ, PPARγ, and AhR proteins relative to the control protein levels. Each column represents the mean ± SEM of three independent experiments (**b**). The blots were stripped and reprobed with an anti-β-actin antibody to control for the amounts of protein loaded onto the gel. *p < 0.05, ***p < 0.001 versus the control

Immunoblot analyses demonstrated that in neurons treated with 10 μM DBP for 6 h, the level of the ERα protein was elevated by 48.57 % compared with that of the control cells. However, after 24 and 48 h of exposure, expression of this protein decreased by 38.93 and 70.92 %, respectively, compared to expression in the control cells (Fig. 5a, b).

A decrease in ERβ protein expression was observed after 6, 24, and 48 h of exposure to 10 μM of DBP (37.40, 60.60, and 61.93 % respectively) compared to that of the control cells.

PPARγ protein expression showed a decrease similar to ERβ expression after 6, 24, and 48 h of exposure (33.19, 41.17, and 53.25 % respectively) compared to that of the control cells.

AhR protein expression began to increase after 3 h by 16.26 % and then significantly decreased after 6, 24, and 48 h (37.28, 30.60, and 19.83 % respectively) compared to that of the control cells.

### Neurotoxic and Apoptotic Effects of DBP in siRNA-Transfected Cells

**Fig. 6:**
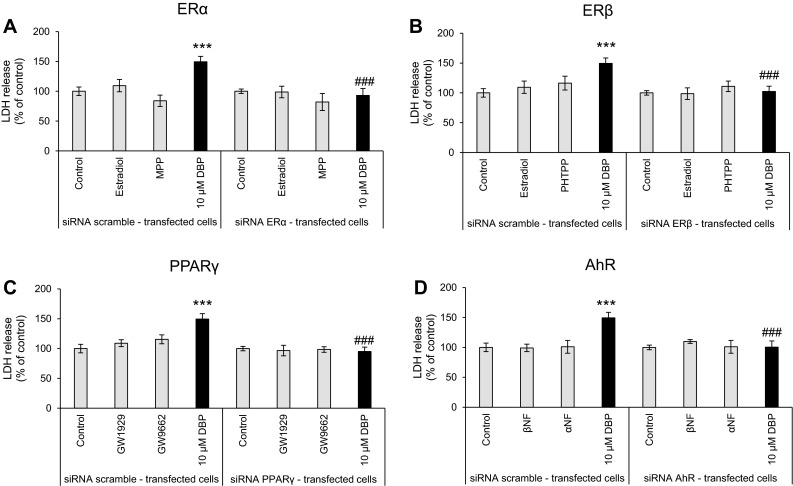
The effect of 10 µM of DBP on LDH release in the negative control siRNA-transfected cells and ERα-specific (**a**), ERβ-specific (**b**), PPARγ-specific (**c**) and AhR-specific (**d**) siRNA-transfected cells. Agonists of ERα (estradiol), ERβ (estradiol), PPARγ (GW1929), and AhR (βNF) were tested. Antagonists of ERα (MPP), ERβ (PHTPP), PPARγ (GW9662), and AhR (αNF) were tested. The data are expressed as the mean ± SEM of four independent experiments, each of which consisted of eight replicates per treatment group. ***p < 0.001 versus the control, ^###^ *p* < 0.001 versus the cells transfected with the negative siRNA

Neocortical neurons were transfected with scramble siRNA and exposed to DBP (10 μM). After 24 h of exposure, a 49.40 % increase in LDH release compared to that of the vehicle control was observed. The effect of DBP on LDH release was reversed by transfection of the neurons with ERα-, ERβ-, PPARγ-, or AhR-specific siRNA (Fig. 6).

**Fig. 7:**
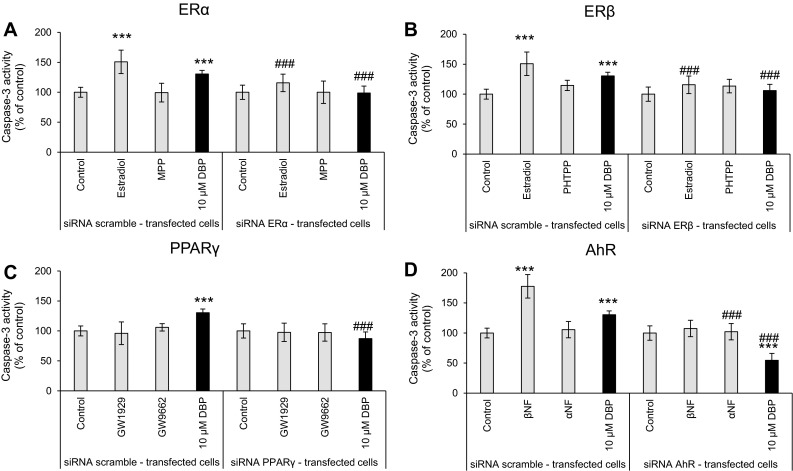
The effect of 10 µM of DBP on caspase-3 activity with negative siRNA-transfected cells and ERα-specific (**a**), ERβ-specific (**b**), PPARγ-specific (**c**) and AhR-specific (**d**) siRNA-transfected cells. Agonists of ERα (estradiol), ERβ (estradiol), PPARγ (GW1929), and AhR (βNF) were tested. Antagonists of ERα (MPP), ERβ (PHTPP), PPARγ (GW9662), and AhR (αNF) were tested. The data are expressed as the mean ± SEM of four independent experiments, each of which consisted of eight replicates per treatment group. ***p < 0.001 versus the control, ^###^ *p* < 0.001 versus the cells transfected with the negative siRNA

Additionally, caspase-3 activity was increased by 30.48 % compared to that of the vehicle control. The effect of DBP on caspase-3 activity was reversed by transfection of the neurons with ERα-, ERβ-, or PPARγ-specific siRNA. Transfection of the neurons with AhR-specific siRNA decreased caspase-3 activity below the control level by 45.52 % (Fig. 7).

The effects of ER (estradiol) or AhR (βNF) agonists were reversed by cell transfection with a specific siRNA.

### Neurotoxic and Apoptotic Effects of DBP with Co-administration of Receptor Antagonists

**Fig. 8:**
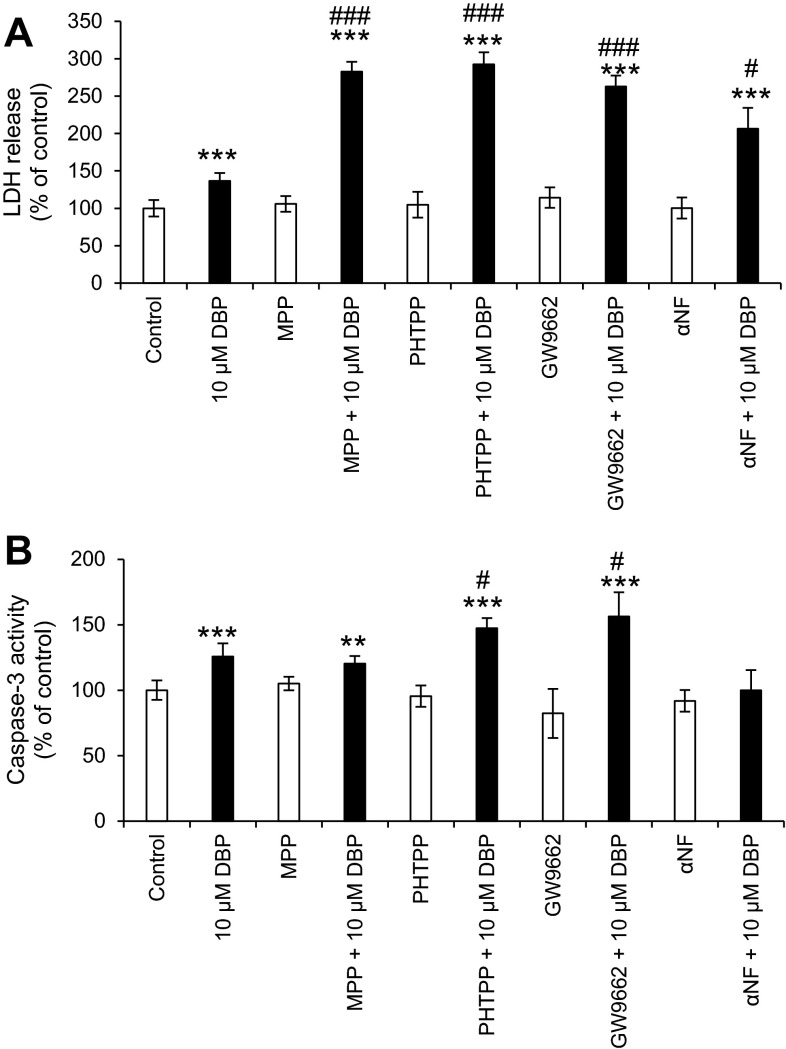
The effect of 10 µM of DBP on LDH release (**a**) and caspase-3 (**b**) activity after co-administration with antagonists of ERα (MPP), ERβ (PHTPP), PPARγ (GW9662), and AhR (αNF) receptors. The data are expressed as the mean ± SEM of four independent experiments, each of which consisted of eight replicates per treatment group. ***p < 0.001 versus the control, ^#^ *p* < 0.05, ^###^ *p* < 0.001; cells treated with 10 µM of DBP versus the cells treated with 10 µM of DBP with co-administration of a receptor antagonist

After 24 h of exposure of neocortical neurons to DBP (10 μM), a 37.73 % increase in LDH release compared to that of the control vehicle was observed. Co-administration of DBP with an ERα antagonist (MPP), ERβ antagonist (PHTPP), PPARγ antagonist (GW9662), or AhR antagonist (αNF) potentiated the LDH release compared to that of the vehicle control by 182.68, 192.46, 162.81, and 106.33 %, respectively (Fig. 8a).

After 24 h of exposure of neocortical neurons to DBP (10 μM), a 25.75 % increase in the caspase-3 activity compared to that of the control vehicle was observed. Co-administration of DBP with an ERα antagonist (MPP), ERβ antagonist (PHTPP) or PPARγ antagonist (GW9662) increased caspase-3 activity compared to that of the vehicle control by 20.37, 47.47, and 56.30 % respectively (Fig. 8b).

## Discussion

This study assessed the neurotoxic and apoptotic effects of DBP in mouse neocortical neurons in primary cultures. DBP stimulated caspase-3 and LDH activities as well as ROS formation in a concentration-(10 nM to 100 µM) and time-dependent (6, 24, 48 h) manner. Interestingly, DBP induced ROS formation at nanomolar concentrations, while it promoted caspase-3 activity and LDH release at micromolar concentrations. The biochemical effects of DBP were accompanied by decreased cell viability and increased apoptotic bodies, as determined by Hoechst 33342 and calcein AM staining. Recently, DBP was shown to activate caspase-3 in the hippocampi of rats that were prenatally exposed to this phthalate (Li et al. 2013). In addition, treatment of adult mice with DBP increased ROS formation and caused oxidative damage in brain tissues (Zuo et al. 2014). Moreover, high micromolar concentrations of DBP were found to cause toxicity in rat embryonic midbrain cell cultures and rat mesencephalic neurospheres (Seek Rhee et al. 2002; Ishido and Suzuki 2014). In contrast to our study, 100 µM DBP did not cause apoptotic effects in the neurospheres as evidenced by TUNEL assays, possibly due to prevalence of DBP-mediated necrosis in these cells. The majority of available studies on phthalate-induced apoptosis and neurotoxicity have focused on the effects of bis(2-ethylhexyl) phthalate (DEHP) and its metabolite mono(2-ethylhexyl) phthalate (MEHP). However, these phthalates have different structures, which may explain their distinct actions in neuronal cells. Similar to the effects of DBP observed in our study, DEHP and MEHP activated caspase-3 in neuro-2a cells and in neurons derived from mouse embryonic stem cells (Lim et al. 2009; Lin et al. 2011). More recently, Peng showed that exposure of adult mice to diisononyl phthalate increased ROS levels and caspase-3 activity and expression in brain tissues (Peng 2015).

In addition to the demonstration of apoptotic and neurotoxic effects of DBP, this study verified the involvement of specific nuclear receptors, such as ERα, ERβ, PPARγ, and AhR, in the DBP-induced effects. In our study, exposure of cells to DBP reduced *ERα* and *PPARγ* mRNA expression levels, which were correlated with decreased protein levels of the receptors. In contrast, treatment with DBP enhanced *AhR* mRNA expression, which was reflected by the increased AhR protein level observed after 3 h of exposure. Interestingly, the DBP-mediated increase in AhR protein was reduced later in the experiment, possibly due to proteasomal degradation of the receptor (Rzemieniec et al. 2015). Chen et al. (2012) showed that AhR protein expression was stimulated by phthalates in human granulosa cells treated with benzyl butyl phthalate (BBP). In our study, a short-term exposure of cells to DBP stimulated *ERβ* mRNA expression. However, a long-term exposure of cells to DBP inhibited this expression, which was correlated with reduced protein levels of the receptor. Our findings are consistent with Li et al. (2014), who showed a down-regulation of ERβ protein expression in the hippocampi of rats that were prenatally exposed to DBP.

Taking into account the DBP-induced alterations in mRNA and protein levels of nuclear receptors, we suggest that AhR is involved in DBP-induced apoptosis and neurotoxicity, whereas the ERs and PPARγ signaling pathways are impaired by the phthalate. To verify this hypothesis, we employed selective receptor antagonists and agonists as well as specific siRNAs. We demonstrated that treatment of the cells with ERα, ERβ or PPARγ antagonists stimulated DBP-induced caspase-3 and LDH activities, which supports our assumption that the effects of DBP are not mediated by ERα, ERβ, and PPARγ. However, this idea was not supported by the experiments using specific siRNAs to silence the receptors. In these experiments, the cells transfected with siRNAs specific for ERα or PPARγ were more resistant to the DBP-induced caspase-3 and LDH effects, which would suggest that both receptors are involved in DBP-induced apoptosis and neurotoxicity. Recently, crosstalk between estrogen receptors and PPARγ has been identified (Chu et al. 2014). These findings suggest that in the present study, ERα silencing stimulated PPARγ expression, while PPARγ silencing stimulated the expression of ERα. Indeed, Ryu et al. (2008) showed that treatment with DBP caused a time- and dose-dependent decrease in the expression of ERα but up-regulated expression of PPARγ in rat testes. Therefore, the reduced effectiveness of DBP observed in the cells with siRNA-silenced ERα could be related to non-specific up-regulation of PPARγ. Similarly, the reduced effectiveness of DBP observed in the cells with siRNA-silenced PPARγ would be related to non-specific up-regulation of ERα. Both receptors are known to have neuroprotective properties; therefore, their presence in the neuronal cells may attenuate the apoptotic and neurotoxic effects of DBP. Additionally, the effects of DBP in siRNA ERβ-transfected cells were attenuated. We suggest that that these results are due to the ability of DBP to act as a weak ERα/β agonist and androgen receptor antagonist, as shown in CHO cells transfected with human ERα or ERβ and in CV-1 cells transfected with ERα (Takeuchi et al. 2005; Shen et al. 2009). In addition, ERβ had neuroprotective effects in primary neocortical, cerebellar, and hippocampal cultures of mouse neurons (Kajta et al. 2013, 2014).

In our study, αNF failed to antagonize DBP-induced LDH release but it showed tendency to inhibit DBP-induced caspase-3 activity. It is possibly because αNF is also a well-documented inhibitor of metabolic reactions that are carried out by the Cyp1a cytochrome family (Bauer et al. 1995). Moreover, we suggest that high concentration of ROS shown in our experiments in response to DBP might have affected expression of AhR-regulated Cyp1a1 that could explain enhanced LDH release in the cells co-treated with DBP and αNF. However, AhR silencing provided evidence that DBP-induced apoptosis and neurotoxicity are mediated by AhR. In the present study, the cells transfected with AhR siRNA were more resistant to the DBP-induced caspase-3 and LDH effects, suggesting the involvement of AhR signaling in DBP-induced effects. Previously, we demonstrated that AhR mediated the apoptotic and neurotoxic effects of DDT and hypoxia (Rzemieniec et al. 2014; Kajta et al. 2014). The only available studies examining the involvement of AhR in the mechanisms of action of phthalates were performed with DEHP and BBP in human breast cancer and endometrial cells and with a luciferase reporter gene assay (Bredhult et al. 2007; Krüger et al. 2008; Hsieh et al. 2012; Mankidy et al. 2013). In the present study, AhR silencing experiments indicated that DBP-induced apoptosis and neurotoxicity are mediated by AhR. These results are consistent with our data on DBP-induced enhancement of AhR mRNA and protein expression. The experiments performed here support our hypothesis that the effects of DBP in neuronal cells are mediated by AhR.

## Conclusion

Our study examined the neurotoxic and apoptotic effects of DBP in mouse neocortical neurons in primary cultures. DBP stimulated caspase-3 and LDH activities as well as ROS formation in a concentration- and time-dependent manner. Interestingly, DBP induced ROS formation at nanomolar concentrations, while it activated caspase-3 activity and LDH release at micromolar concentrations. We demonstrated that the DBP mechanism of action involves ERα, ERβ, PPARγ, and AhR. Our study showed that AhR mediates DBP-induced apoptosis and neurotoxicity, whereas the ERs and PPARγ signaling pathways are impaired by the phthalate. However, it is also possible that DBP activates other molecular signaling pathways. Therefore, further studies on the mechanisms underlying the effects of DBP on the nervous system are needed.

## Abbreviations

AhR: Aryl hydrocarbon receptor
DBP: Dibutyl phthalate
DMSO: Dimethyl sulfoxide
ERα: Estrogen receptor alpha
ERβ: Estrogen receptor beta
FBS: Fetal bovine serum
H_2_DCFDA: 2,7′-Dichlorodihydrofluorescein diacetate
LDH: Lactate dehydrogenase
MPP: 1,3-Bis(4-hydroxyphenyl)-4-methyl-5-[4-(2-piperidinylethoxy)phenol]-1h-pyrazole dihydrochloride
PBS: Phosphate buffered saline
PHTPP: 4-[2-Phenyl-5,7-bis(trifluoromethyl)pyrazolo[1,5-a]pyrimidin-3-yl]phenol
PPARγ: Peroxisome proliferator-activated receptor gamma
ROS: Reactive oxygen species
αNF: α-Naphthoflavone
βNF: β-Naphthoflavone
